# Correction: p190 RhoGAP promotes contact inhibition in epithelial cells by repressing YAP activity

**DOI:** 10.1083/JCB.20171005807172018c

**Published:** 2018-09-03

**Authors:** Scott R. Frank, Clemens P. Köllmann, Phi Luong, Giorgio G. Galli, Lihua Zou, André Bernards, Gad Getz, Raffaele A. Calogero, Morten Frödin, Steen H. Hansen

June 6, 2018. 10.1083/jcb.201710058.

Fig. 6 C inadvertently contained an image duplication of MDCK cells expressing EGFP instead of EGFP-RhoA(F30L). The authors apologize for this error, which does not affect the conclusions of Fig. 6 C or any other data presented in the paper. Below is the corrected figure, which properly shows cells expressing EGPF-RhoA(F30L) in the right panel of Fig. 6 C:

**Figure:**
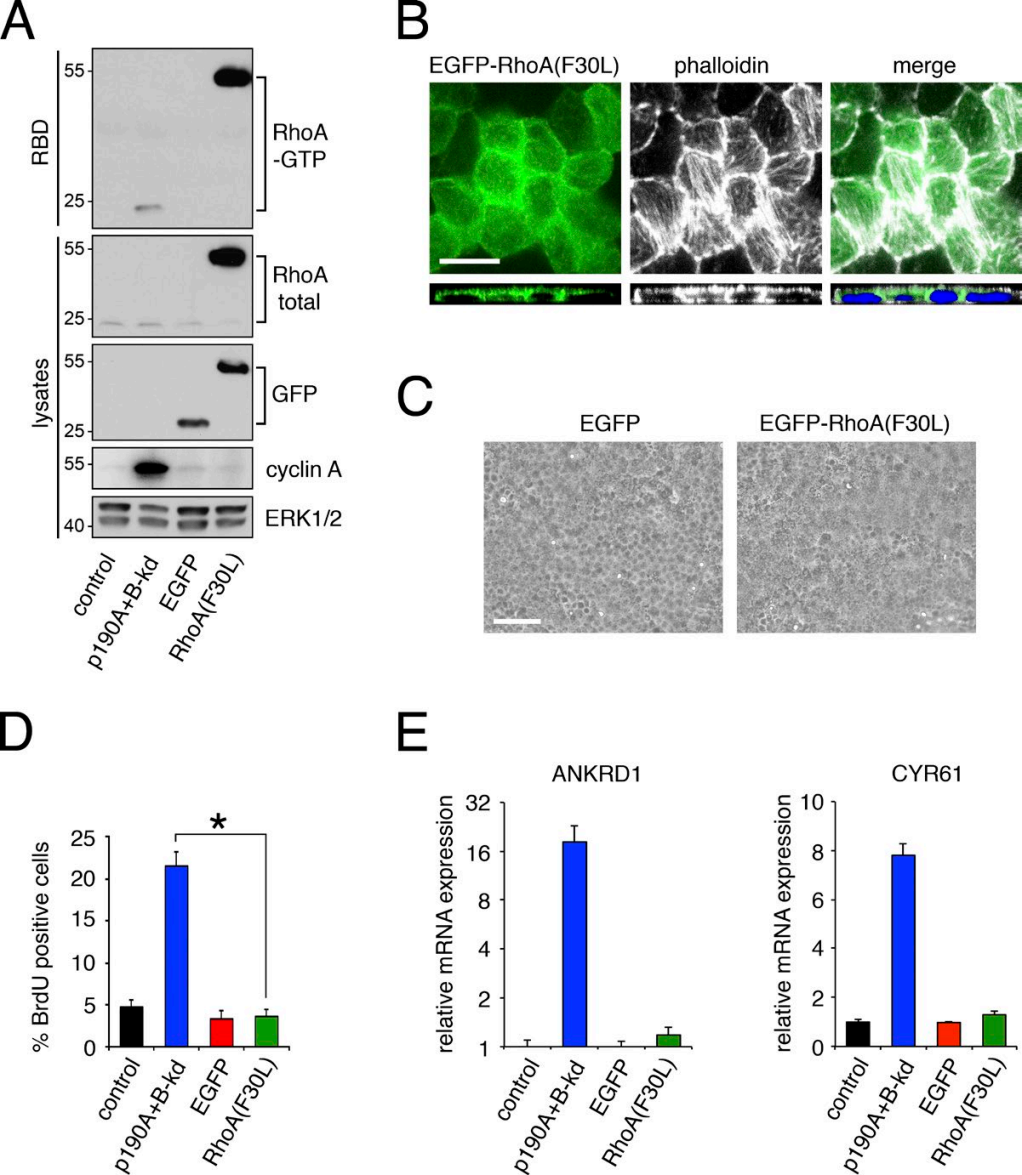


Both the HTML and PDF versions of the article have been corrected.

